# Association between blood glucose variability and coronary plaque instability in patients with acute coronary syndromes

**DOI:** 10.1186/s12933-015-0275-3

**Published:** 2015-08-20

**Authors:** Kozo Okada, Kiyoshi Hibi, Masaomi Gohbara, Shunsuke Kataoka, Keiko Takano, Eiichi Akiyama, Yasushi Matsuzawa, Kenichiro Saka, Nobuhiko Maejima, Mitsuaki Endo, Noriaki Iwahashi, Kengo Tsukahara, Masami Kosuge, Toshiaki Ebina, Peter J. Fitzgerald, Yasuhiro Honda, Satoshi Umemura, Kazuo Kimura

**Affiliations:** Division of Cardiology, Yokohama City University Medical Center, 4-57 Urafune-cho, Minami-ku, Yokohama, 232-0024 Japan; Division of Cardiovascular Medicine, Stanford University Medical Center, Stanford, USA; Department of Medical Science and Cardiorenal Medicine, Yokohama City University Graduate School of Medicine, Yokohama, Japan

**Keywords:** Glucose variability, Vulnerable plaque, Acute coronary syndrome, IB-IVUS

## Abstract

**Background:**

Blood glucose variability is receiving considerable attention as a new risk factor for coronary artery disease. This study aimed to investigate the association between blood glucose variability and coronary plaque tissue characteristics.

**Methods:**

In 57 patients with acute coronary syndrome, integrated backscatter intravascular ultrasound (IB-IVUS) and gray-scale IVUS were performed before balloon dilatation or stent implantation in the culprit vessels. Standard IVUS indices were evaluated for volume index (volume/length), and plaque components were measured by IB-IVUS for percent tissue volume. In addition to conventional glucose indicators, blood glucose variability in a stable state was determined by calculating the mean amplitude of glycemic excursions (MAGE) using a continuous glucose monitoring system.

**Results:**

Higher MAGE values were significantly correlated with larger percent plaque volumes (r = 0.32, p = 0.015), and increased lipid (r = 0.44, p = 0.0006) and decreased fibrous (r = −0.45, p = 0.0005) plaque components. In contrast, HbA1c or fasting plasma glucose values were not significantly correlated with plaque volumes and percent plaque components. Homeostasis model assessment of insulin resistance values were positively correlated with vessel (r = 0.35, p = 0.007) and plaque (r = 0.27, p = 0.046) volumes, but not with percent plaque components. In multiple regression analysis, higher MAGE values were independently associated with increased lipid (β = 0.80, p = 0.0035) and decreased fibrous (β = -0.79, p = 0.0034) contents in coronary plaques.

**Conclusions:**

Among all glucose indicators studied, only higher blood glucose variability was an independent determinant of increased lipid and decreased fibrous contents with larger plaque burden, suggesting blood glucose variability as one of the important factors related to coronary plaque vulnerability.

## Background

Glycemic disorder is an important risk factor of coronary plaque progression and instability, and subsequent acute coronary syndromes (ACS) [1–7]. While previous studies focused primarily on hemoglobin A1c (HbA1c), fasting plasma glucose (FPG), homeostasis model assessment of insulin resistance (HOMA-IR), advanced glycation end products (AGEs), and glucagon-like peptide 1 (GLP-1) to assess the severity of diabetes-related vascular complications [1–5], blood glucose variability has been recognized as another important measure in recent investigations [8–10]. Continuous glucose monitoring system (CGMS) can directly visualize blood glucose variability, offering a more sensitive method to detect the variety of glycemic disorder compared to other conventional glucose indicators (HbA1c, FPG, HOMA-IR) [11].

Previous CGMS studies of patients with diabetes mellitus (DM) have shown that blood glucose variability was more strongly associated with atherogenic factors, such as oxidative stress, endothelial dysfunction and inflammation compared to sustained hyperglycemia represented as HbA1c and FPG [8, 9, 12]. Hence, it is reasonable to hypothesize that the assessment of blood glucose variability using CGMS may more accurately measure the severity of diabetes-related vascular disease (in particular, atherosclerotic plaques) compared to conventional glucose indicators. Therefore, the aims of this study are to characterize blood glucose variability as measured by CGMS and to investigate its association with coronary tissue characteristics in patients with ACS.

## Methods

### Study population

This was a prospective, observational study of ACS patients admitted to Yokohama City University Medical Center. ACS patients who underwent both percutaneous coronary intervention (PCI) with integrated backscatter (IB) intravascular ultrasound (IVUS) guidance in the culprit vessel and CGMS measurement were eligible for enrollment. Patients with renal dysfunction (serum creatinine >2.0 mg/dl at admission) or receiving insulin (for both type 1 and type 2 DM) were excluded. ACS consisted of ST-segment elevation myocardial infarction (STEMI) and non ST-segment elevation ACS (NSTE-ACS). STEMI was defined as the presence of anginal symptoms (>20 min) associated with electrocardiographic ST-segment elevation of at least 0.1 mV in two or more limb leads or at least 0.2 mV in two or more precordial leads, and a rise in cardiac-specific troponin I values. NSTE-ACS included non ST-segment elevation myocardial infarction (NSTEMI) and unstable angina pectoris. NSTEMI was defined as ischemic symptoms in the absence of ST elevation on electrocardiogram with elevated cardiac-specific troponin I values. Unstable angina pectoris was defined as having newly developed and accelerated chest symptoms on exertion or rest angina without a significant rise in cardiac-specific troponin I values. DM was determined by the following criteria: medical history, FPG value of ≥126 mg/dl; casual plasma glucose value of ≥200 mg/dl; or diabetic pattern based on 75-g oral glucose tolerance tests (OGTT). OGTT was performed in a stable condition (at 12 ± 5 days) during hospital admission. The study protocol was approved by the Institutional Review Board at Yokohama City University Medical Center, and every patient provided written informed consent.

### CGMS

All CGMS monitoring was performed in a stable condition without any treatment with anti-diabetic drugs during hospital admission (at 10 ± 6 days) to minimize the influence of ACS and anti-diabetic medications on CGMS monitoring: inflammation caused by ACS had peaked; and patients had regular meals and were ambulatory [13]. Study patients were equipped with a fourth-generation CGMS (iPro2, Medtronic, USA) and were monitored for 24 consecutive hours. Patients received three energy-controlled meals per day during CGMS monitoring. A CGMS sensor was inserted into the subcutaneous abdominal fat tissue. Ipro2 uses a retrospective algorithm to convert sensor signals to glucose levels based on self-monitoring of capillary blood glucose readings; therefore, blood glucose values were checked at least four times per day using the finger-stick test. Blood glucose variability was determined by the mean amplitude of glycemic excursions (MAGE) based on the CGMS data [12]. MAGE values were calculated by measuring the arithmetic mean of differences between consecutive peaks and nadirs, providing that the differences were greater than one standard deviation (SD) of the mean blood glucose value; measurements in the peak-to-nadir or nadir-to-peak directions were determined by the first qualifying excursion. In this study, significant hyperglycemia and significant hypoglycemia were defined as a blood glucose value of ≥200 and <60 mg/dl, respectively [14, 15].

### Laboratory evaluation

In addition to CGMS parameters, conventional glucose indicators (HbA1c, FPG, HOMA-IR), lipid variables [total cholesterol (TC), low-density lipoprotein cholesterol (LDL-C), high-density lipoprotein cholesterol (HDL-C), triglycerides], high-sensitive C-reactive protein (hs-CRP) values, and estimated glomerular filtration rate (eGFR) were evaluated. While HbA1c, lipid variables and eGFR values were measured at admission, FPG and HOMA-IR (at 12 ± 5 days), and hs-CRP (at 7 days) values were measured in a stable condition during hospital admission. eGFR values were determined from serum creatinine values, using the prediction equation proposed by the Japanese Society of Nephrology. HOMA-IR values were mathematically calculated by the formula: [fasting plasma glucose (FPG, mg/dl) × fasting immunoreactive insulin (IRI, μU/ml)/405] [2]. Urinary liver-type fatty acid-binding protein (L-FABP) values in 52 patients were also measured in the first morning urine on the day after admission as an indirect marker of oxidative stress [16].

### IVUS procedure

Coronary angiography was performed via the femoral or radial approach. Heparin (5000–10,000 units) was administered intravenously before PCI, and periprocedural intravenous heparin was administered to maintain an activated clotting time ≥250 s. Intracoronary isosorbide dinitrate (ISDN) was also administered before angiography to prevent coronary artery spasm. IVUS imaging was performed using a mechanical IVUS system with a 40-MHz, 5 Fr imaging catheter (VISIWAVE with ViewIT. Terumo, Tokyo, Japan) before balloon dilation or stent implantation [2, 4, 6, 17]. Thrombus aspiration was performed prior to IVUS as necessary. After ISDN administration (2–3 mg), the catheter was advanced sufficiently distal to the culprit lesion, and automated pullback was then performed at 0.5 mm/s. Images were recorded for offline analysis of each culprit vessel.

### IVUS analysis

Intravascular ultrasound analysis was performed with a validated quantitative IVUS analysis system (VISIATLAS, Terumo, Tokyo, Japan) [18] by an experienced investigator, blinded to clinical information. Vessel, lumen and plaque (vessel minus lumen) areas were manually traced at 1-mm intervals throughout each entire culprit vessel (average length analyzed: 66 ± 26 mm), and the interpolated measurements of the remaining frames were automatically generated [19]. Vessel, lumen and plaque volumes were calculated using Simpson’s method and standardized as volume index (volume/analyzed length, mm^3^/mm). Percent plaque volume was calculated as (plaque volume/vessel volume) × 100 (%). Remodeling index was calculated mathematically as vessel area at the minimum lumen area site divided by the average vessel area of the proximal and distal reference sites [19]. IB data for each tissue component were calculated as average power levels using a fast Fourier transform, measured in decibels, of the frequency component of backscattered signals from a small volume of tissue. IB-IVUS analysis classified the color-coded tissue into four major components: blue (lipid); green (fibrosis); yellow (dense fibrosis); red (calcification) [2]. Quantitative volumetric IB-IVUS analyses were performed to calculate lipid, fibrosis, dense fibrosis, and calcification volumes from the sum of lipid, fibrosis, dense fibrosis, and calcification areas in each cross-sectional area at 1-mm intervals for the IB-IVUS images. Percentages of plaque volume were automatically calculated as each plaque component volume/plaque volume × 100 (%). Intra- or interobserver intra-class correlation coefficients (ICC) for the vessel, lumen, and plaque areas were 0.999 and 0.999, 0.996 and 0.993, and 0.993 and 0.991, respectively. The intra- and interobserver ICC for the percent lipid and fibrous areas were 0.997 and 0.996, and 0.996 and 0.995, as we previously reported [18].

### Statistical analysis

Data are expressed as frequencies and percentages for category variables, and as mean ± SD for continuous variables. Categorical comparisons were performed using a Chi square test or Fisher’s exact test. Continuous values were compared by using unpaired t-test, Mann–Whitney U test, or one-way analyses of variance, as appropriate. Correlation between continuous variables was evaluated by using linear regression analysis. Multiple linear regression analysis was performed to determine the factors associated with percent lipid and fibrous contents of coronary plaques. Factors analyzed were: age; sex; body mass index; medical history (diabetes, hyperlipidemia, hypertension, current smoking, type of ACS, culprit vessel, presence of mutivessel coronary disease); pre-treatment with statins or anti-diabetic drugs before admission; conventional glucose indicators (HbA1c, FPG, HOMA-IR); CGMS indices (MAGEA, mean, maximum, and minimum blood glucose values, episodes of significant hyperglycemia ≥200 mg/dl and hypoglycemia <60 mg/dl); lipid variables (TC, LDL-C, HDL-C, triglycerides); hs-CRP; and eGFR values. Variables with p-value ≤0.15 on univariable analysis were entered into the multivariable model. A p-value of <0.05 was considered statistically significant. Statistical calculations were performed with JMP^®^ 10 (SAS Institute Inc., Cary, NC, USA).

## Results

### Patient characteristics

A total of 76 consecutive patients with ACS who met the inclusion and exclusion criteria and underwent both IB-IVUS and CGMS examinations were enrolled in this study. Nineteen were excluded because of poor-quality IVUS images (5 patients), stent thrombosis (1 patient), no IVUS imaging before PCI (5 patients), or insufficient CGMS data for analysis (8 patients). As a result, 57 data from 57 patients were analyzed. As seen in Table 1, the average age was 65 ± 12 years, and the majority of patients were men. The proportion of STEMI was 77.2 % and DM was 49.1 %. HbA1c and FPG values were 6.1 ± 1.0 % and 109 ± 17 mg/dl, respectively. HOMA-IR values (insulin resistance) were 2.1 ± 1.4. The percentage of patients treated with statins before admission was 21.1 % and LDL-C values at admission were 128 ± 36 mg/dl.

**Table 1:** Clinical characteristics and CGMS parameters

Variables	All (n = 57)
Age (years)	65 ± 12
Male (%)	78.9
Body mass index (kg/m^2^)	24.2 ± 4.1
STEMI (%)	77.2
Multivessel disease (%)	35.1
Culprit vessel	
Left anterior descending artery (%)	52.6
Left circumflex coronary artery (%)	14.0
Right coronary artery (%)	33.3
Current smoking (%)	54.4
Hypertension (%)	61.4
Diabetes mellitus (%)	49.1
Lipid parameters at admission	
Total cholesterol (mg/dl)	201 ± 37
LDL-C (mg/dl)	128 ± 36
HDL-C (mg/dl)	46 ± 12
Triglycerides (mg/dl)	129 ± 96
eGFR at admission (ml/min/1.73 m^2^)	75.2 ± 18.2
Urinary L-FABP (μg/g creatinine)	20.3 ± 58.0
hs-CRP at 7 days after admission (mg/dl)	1.56 ± 1.72
Medications before admission	
Statin (%)	21.1
ACEI/ARB (%)	21.1
Oral anti-diabetic drugs (%)	17.5
Conventional glucose indicators	
HbA1c at admission (%)	6.1 ± 1.0
FPG^a^ (mg/dl)	109 ± 17
HOMA-IR^a^	2.1 ± 1.4
CGMS parameters^a^	
Maximum blood glucose (mg/dl)	184 ± 48
Minimum blood glucose (mg/dl)	90 ± 21
Mean blood glucose (mg/dl)	126 ± 27
SD around mean blood glucose (mg/dl)	22 ± 12
MAGE (blood glucose variability) (mg/dl)	48 ± 26
Hyperglycemia ≥200 mg/dl (%)	29.8
Hypoglycemia <60 mg/dl (%)	7.0

*ACEI/ARB* angiotensin-converting enzyme inhibitor and/or angiotensin II receptor blocker, *CGMS* continuous glucose monitoring system, *eGFR* estimated glomerular filtration ratio, *FPG* fasting plasma glucose, *HDL*-*C* high-density lipoprotein cholesterol, *HOMA*-*IR* homeostasis model assessment of insulin resistance, *LDL*-*C* low-density lipoprotein cholesterol, *MAGE* mean amplitude of glucose excursion, *STEMI* ST-segment elevation myocardial infarction, *L*-*FABP* liver-type fatty acid-binding protein (in the first morning urine on the day after admission)

^a^Measured in a stable condition during hospital admission

### CGMS parameters

**Fig. 1:**
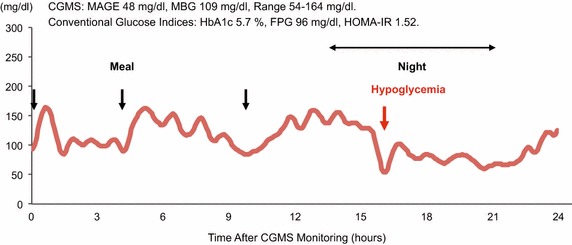
Representative case of CGMS monitoring. Patient was a 59-year-old man who was diagnosed with STEMI. He had medical history of hypertension, hyperlipidemia and current smoking, and was diagnosed with impaired glucose tolerance on OGTT during hospital admission. Conventional glucose indicators were within normal range: however, CGMS monitoring revealed sustained postprandial hyperglycemia that was more prominent toward the evening meal, and significant hypoglycemia at night. *MBG* mean blood glucose, *FPG* fasting plasma glucose

Continuous glucose monitoring system data include a mean blood glucose value of 126 ± 27 mg/dl and MAGE (blood glucose variability) value of 48 ± 26 mg/dl (Table 1). During CGMS monitoring, blood glucose values largely remained within the range of 90–200 mg/dl, as recommended by the guideline [14]; however, various glucose variability patterns were seen, irrespective of glucose metabolism (normal glucose tolerance, impaired glucose tolerance, type 2 DM). In addition, asymptomatic hyperglycemia and hypoglycemia were found in some patients. Significant hyperglycemia (≥200 mg/dl) was seen in 29.8 % of the patients. Significant hypoglycemia (<60 mg/dl) was found in 7.0 % of patients. The incidence of hypoglycemia did not differ significantly between patients with and without DM (7.1 vs. 6.9 %, p = 0.97), and all hypoglycemia were seen in patients without treatment with anti-diabetic drugs before admission. Figure 1 shows a representative case of CGMS monitoring where sustained postprandial hyperglycemia was more prominent toward the evening meal and significant hypoglycemia (54 mg/dl) was found at night despite conventional glucose indicators being within normal range.

In terms of relationships among CGMS parameters, MAGE values were significantly correlated with maximum blood glucose values (r = 0.88, p < 0.0001) and mean blood glucose values (r = 0.55, p < 0.0001), but not significantly with minimum blood glucose values (r = 0.05, p = 0.70). When study patients were divided into two groups based on the median value of MAGE, proportions of significant hyperglycemia (≥200 mg/dl) (57.1 vs. 3.5 %, p < 0.0001) and hypoglycemia (<60 mg/dl) (14.3 vs. 0 %, p = 0.014) were found to be greater in patients with higher blood glucose variability compared to those with lower blood glucose variability.

### Relationships between conventional glucose indicators and CGMS parameters

HbA1c and FPG were positively correlated with maximum blood glucose, minimum blood glucose, mean blood glucose, and MAGE values, while HOMA-IR values were not (Table 2). In the categorical analyses (higher or lower than each median value of conventional glucose indicators), significant hyperglycemia (≥200 mg/dl) was more frequent in patients with higher HbA1c (53.6 vs. 6.9 %, p < 0.0001) or higher FPG (50.0 vs. 10.3 %, p = 0.0007) values compared to those with lower HbA1c or FPG values, whereas the incidence of significant hypoglycemia (<60 mg/dl) did not differ significantly between the higher and lower groups (3.6 vs. 10.3 %, p = 0.31 for both HbA1c and FPG). There was also no significant difference in hyperglycemia (28.6 vs. 32.1 %, p = 0.77) or hypoglycemia (3.6 vs. 10.7 %, p = 0.29) between patients with higher and lower HOMA-IR values.

**Table 2:** Correlations between conventional glucose indicators and CGMS parameters

Variables (mg/dl)	HbA1c (%)	FPG (mg/dl)	HOMA-IR
MAGE (glucose variability)	r = 0.36 p = 0.0065	r = 0.47, p = 0.0002	r = −0.07, p = 0.59
Mean blood glucose	r = 0.63, p < 0.0001	r = 0.84, p < 0.0001	r = −0.0003, p = 0.998
Maximum blood glucose	r = 0.48, p = 0.0002	r = 0.69, p < 0.0001	r = −0.05, p = 0.73
Minimum blood glucose	r = 0.57, p < 0.0001	r = 0.60, p < 0.0001	r = 0.07, p = 0.61

Abbreviations shown in Table 1

### Relationships of glucose indicators to oxidative stress and inflammation

Urinary L-FABP values were significantly correlated with MAGE values (r = 0.37, p = 0.008) and tended to correlate with HOMA-IR values (r = 0.27, p = 0.054), but not with HbA1c (r = 0.05, p = 0.74) or FPG values (r = 0.09, p = 0.53). On the other hand, hs-CRP values tended to correlate with MAGE values (r = 0.25, p = 0.066), but not with conventional glucose indicators (HbA1c: r = −0.05, p = 0.71; FPG: r = 0.004, p = 0.98; HOMA-IR: r = −0.04, p = 0.77).

### Factors associated with gray-scale IVUS indices

**Fig. 2:**
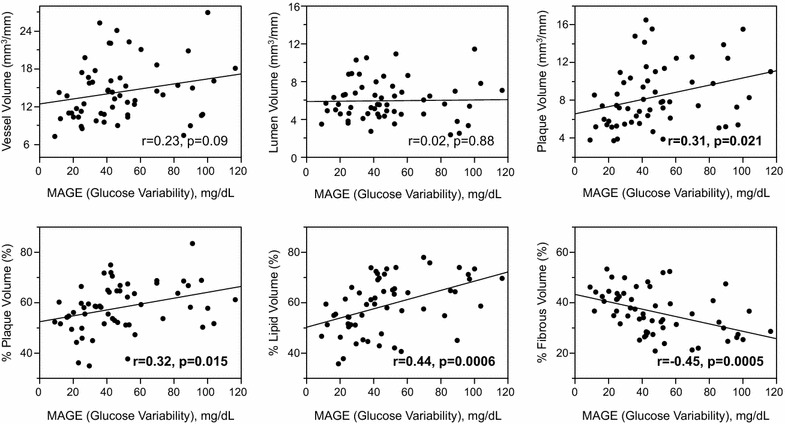
Correlations between glucose variability and IVUS indices

**Fig. 3:**
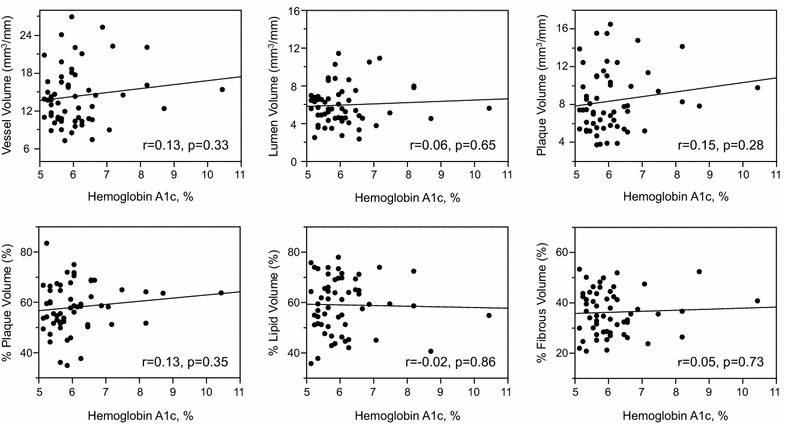
Correlations between hemoglobin A1c and IVUS indices

**Fig. 4:**
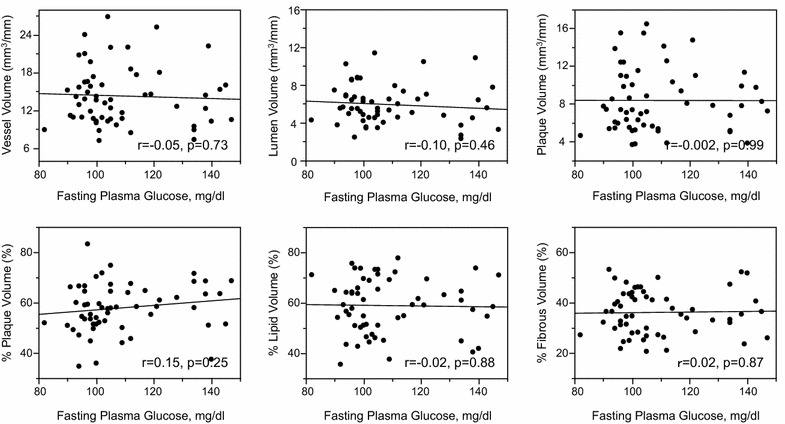
Correlations between fasting plasma glucose and IVUS indices

**Fig. 5:**
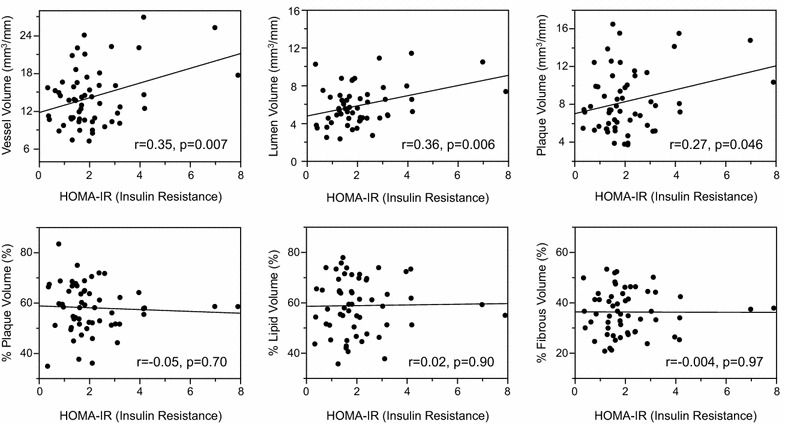
Correlations between HOMA-IR and IVUS indices

Mean amplitude of glycemic excursions (blood glucose variability) values were positively correlated with plaque volumes, percent plaque volumes and remodeling index (r = 0.27, p = 0.046), and tended to correlate with vessel volumes (Fig. 2). In contrast, mean blood glucose values as measured by CGMS (vessel volumes: r = −0.09, p = 0.51; lumen volumes: r = −0.07, p = 0.58; plaque volumes: r = −0.08, p = 0.56; percent plaque volumes: r = 0.06, p = 0.63; remodeling index: r = 0.20, p = 0.15) or HbA1c values (Fig. 3; remodeling index: r = 0.21, p = 0.13) were not correlated with any gray-scale IVUS indices. FPG values were correlated with remodeling index (r = 0.33, p = 0.013), but not with other IVUS indices (Fig. 4). HOMA-IR values were correlated with vessel, lumen and plaque volumes, but not with percent plaque volumes (Fig. 5) or remodeling index (r = −0.05, p = 0.71). Patients with significant hyperglycemia (≥200 mg/dl) tended to have greater plaque burden compared to those without (61.6 ± 10.4 vs. 56.3 ± 8.9 %, p = 0.053). Patient with significant hypoglycemia (<60 mg/dl) also had greater percent plaque volume compared to those without (69.4 ± 9.6 vs. 57.0 ± 9.1 %, p = 0.011). While MAGE values were significantly higher in patients with DM compared to those without (60 ± 26 vs. 37 ± 22 mg/dl, p = 0.0003), gray-scale IVUS indices were comparable between the two groups (Table 3). LDL-C or hs-CRP value was not significantly correlated with any gray-scale IVUS indices.

**Table 3:** Gray-scale IVUS and IB-IVUS indices

Variables	All (n = 57)	DM (n = 28)	Non DM (n = 29)	p value*
Vessel volume (mm^3^/mm)	14.3 ± 4.6	14.4 ± 4.7	14.2 ± 4.7	0.89
Lumen volume (mm^3^/mm)	5.9 ± 2.1	5.8 ± 2.1	6.0 ± 2.1	0.68
Plaque volume (mm^3^/mm)	8.4 ± 3.3	8.6 ± 3.1	8.2 ± 3.5	0.51
Percent plaque volume (%)	57.9 ± 9.6	59.3 ± 8.9	56.6 ± 10.1	0.29
Remodeling index	1.08 ± 0.17	1.10 ± 0.16	1.05 ± 0.17	0.27
Lipid volume (mm^3^/mm)	5.1 ± 2.6	5.3 ± 2.5	4.9 ± 2.7	0.45
Fibrous volume (mm^3^/mm)	2.9 ± 0.9	2.9 ± 0.9	2.9 ± 1.0	0.96
Percent lipid volume (%)	58.9 ± 10.9	59.7 ± 11.6	58.1 ± 10.4	0.57
Percent fibrous volume (%)	36.1 ± 8.7	35.2 ± 8.9	37.0 ± 8.5	0.44

*DM* diabetes mellitus

* p-values for DM vs. non-DM

### Factors associated with percent lipid and fibrous volumes

Mean amplitude of glycemic excursions values were positively correlated with percent lipid volumes (%LV) and negatively with percent fibrous volumes (%FV) (Fig. 2). In contrast, conventional glucose indicators (HbA1c, FPG, HOMA-IR), and mean blood glucose values as measured by CGMS were not significantly correlated with %LV or %FV (Figs. 3, 4, 5; Tables 4, 5). Patients with significant hyperglycemia tended to have higher %LV (62.4 ± 11.5 vs. 57.4 ± 10.5 %, p = 0.12) and lower %FV (33.3 ± 9.6 vs. 37.3 ± 8.1 %, p = 0.12) compared to those without. Patients with significant hypoglycemia also had numerically higher %LV (66.3 ± 6.9 vs. 58.3 ± 11.0 %, p = 0.16) and lower %FV (30.3 ± 5.9 vs. 36.5 ± 8.8 %, p = 0.17) compared to those without, yet the differences did not reach statistical significance. On the other hand, the presence of DM, treatment with statins or anti-diabetic drugs before admission, LDL-C or hs-CRP value was not associated with plaque components (Tables 4, 5). Although MAGE values were significantly higher in patients with STEMI compared to those with NSTE-ACS (53 ± 27 vs. 34 ± 17 mg/dl, p = 0.03), the type of ACS (STEMI or NSTE-ACS) was not associated with plaque components (Tables 4, 5).

**Table 4:** Factors associated with percent lipid volumes

Variables	Univariable		Multivariable	
	r	p value	β	p value
Age (years)	0.26	0.055	0.13	0.30
STEMI	0.05	0.73		
Statin before admission	−0.07	0.57		
Anti-diabetic drugs before admission	0.18	0.18		
LDL-C (mg/dl)	−0.17	0.21		
HDL-C (mg/dl)	0.24	0.07	0.14	0.26
hs-CRP (mg/dl)	0.16	0.24		
eGFR (ml/min/1.73 m^2^)	−0.19	0.16		
HbA1c (%)	−0.02	0.86		
FPG (mg/dl)	−0.02	0.88		
HOMA-IR	0.02	0.90		
MAGE (glucose variability) (mg/dl)	0.44	0.0006	0.80	0.0035
Mean blood glucose (mg/dl)	0.001	0.99		
Maximum blood glucose (mg/dl)	0.29	0.03	−0.25	0.44
Hyperglycemia (≥200 mg/dl)	0.21	0.12	−0.25	0.28
Hypoglycemia (<60 mg/dl)	0.19	0.16		

Abbreviations shown in Table 1

**Table 5:** Factors associated with percent fibrous volume

Variables	Univariable		Multivariable	
	r	p value	β	p value
Age (years)	−0.27	0.04	−0.14	0.25
STEMI	−0.03	0.80		
Statin before admission	0.08	0.56		
Anti-diabetic drugs before admission	−0.19	0.16		
LDL-C (mg/dl)	0.18	0.19		
HDL-C (mg/dl)	−0.27	0.04	−0.17	0.17
hs-CRP (mg/dl)	−0.19	0.153		
eGFR (ml/min/1.73 m^2^)	0.16	0.25		
HbA1c (%)	0.05	0.73		
FPG (mg/dl)	0.02	0.87		
HOMA-IR	−0.004	0.97		
MAGE (glucose variability) (mg/dl)	−0.45	0.0005	−0.79	0.0034
Mean blood glucose (mg/dl)	0.002	0.99		
Maximum blood glucose (mg/dl)	−0.29	0.03	0.24	0.44
Hyperglycemia (≥200 mg/dl)	−0.21	0.12	0.26	0.27
Hypoglycemia (<60 mg/dl)	−0.18	0.17		

Abbreviations shown in Table 1

Significant correlations of MAGE values with plaque instability were found in the subgroup analyses (Table 6). In addition, significant correlations of MAGE values with plaque instability were seen when we excluded the 10-mm length segment centered at the minimum lumen area (MLA) site in the culprit vessel from the original analyses to minimize influence of thrombus around the culprit lesion (%LV: r = 0.41, p = 0.0014;  %FV: r = −0.41, p = 0.0017). In the multiple regression analysis including all variables with p-value of ≤0.15 on univariable analysis, only MAGE values were independently associated with both increased %LV and decreased %FV (Tables 4, 5). Higher MAGE value was also an independent risk factor for increased %LV and decreased %FV in multivariable analysis including LDL-C (%LV: β = 0.84, p = 0.0026; %FV: β = −0.83, p = 0.0025) or urinary L-FABP (%LV: β = 0.63, p = 0.026; %FV: β = −0.62, p = 0.026) in the original model, whereas LDL-C and urinary L-FABP values were not significantly associated with plaque components.

**Table 6:** Subgroup analyses of relationships between blood glucose variability and percent lipid and fibrous volume

Subgroup	Percent lipid volumes	Percent fibrous volumes
DM	r = 0.29, p = 0.13	r = −0.26, p = 0.18
Non DM	r = 0.67, p < 0.0001	r = −0.68, p < 0.0001
STEMI	r = 0.41, p = 0.006	r = −0.41, p = 0.006
NSTE-ACS	r = 0.73, p = 0.005	r = −0.76, p = 0.003
No treatment with statins before admission	r = 0.50, p = 0.0005	r = −0.51, p = 0.0004
No treatment with anti-diabetic drugs before admission	r = 0.50, p = 0.0004	r = −0.50, p = 0.0004
LDL-C ≥140 mg/dl	r = 0.48, p = 0.02	r = −0.51, p = 0.01
LDL-C <140 mg/dl	r = 0.44, p = 0.01	r = −0.42, p = 0.01

Abbreviations shown in Table 1

## Discussion

The main findings of this study are: (1) higher blood glucose variability was more strongly associated with increased lipid and decreased fibrous contents with larger plaque burden and higher remodeling index in the culprit vessels of ACS as compared to conventional glucose indicators (HbA1c, FPS, HOMA-IR); and (2) higher blood glucose variability was an independent risk factor for plaque instability. Higher blood glucose variability was also more closely linked with the markers of oxidative stress and inflammation as compared to conventional glucose indicators. Our findings suggest the potential utility of the assessment of blood glucose variability using CGMS in the prediction of vulnerable plaques, and indicate the underlying mechanisms of the association between higher blood glucose variability and coronary plaque instability.

### Vulnerable plaque as assessed by IB-IVUS

Previous studies have demonstrated that disruption or erosion of vulnerable plaques and subsequent thrombus formation are the most frequent causes of ACS [20]. Lipid incorporation into the arterial wall is a key player in the initiation and progression of atherosclerosis, and lipid-rich plaque is associated with coronary plaque instability, an important determinant of spontaneous plaque rupture [2, 6, 21]. In the pathologic study assessing vulnerable plaque after ACS, larger lipid core is recognized as the marker for plaque vulnerability [21]. In the IB-IVUS study evaluating plaque morphology before the occurrence of ACS [22], the percent lipid area was greater and percent fibrous area was smaller in coronary plaques that caused ACS than in those without ACS. Another study has reported that higher percent lipid area and lower percent fibrous area were associated with thin-cap fibroatheroma as evaluated by optical coherence tomography (OCT) [23]. These findings suggest that increased lipid and decreased fibrous contents in coronary plaques can be good indicators of vulnerable plaques.

### Blood glucose variability

In the present study, we found that among all the glucose indicators studied, only higher blood glucose variability (MAGE values) was an independent determinant of plaque instability in the culprit vessel of ACS. Previous studies have reported that blood glucose variability as measured by CGMS was more strongly associated with atherogenic factors, such as oxidative stress, inflammation, and endothelial dysfunction compared to sustained hyperglycemia represented as HbA1c and FPG [8, 9, 12, 24]. All these factors link with coronary plaque instability. In particular, oxidative stress plays a pivotal role in diabetic vascular disease, and modulates the expression of matrix metalloproteinases (MMPs) and the formation of oxidative LDL [25, 26]. MMPs may contribute to plaque vulnerability by degrading the components of the fibrous cap [25]. Oxidative LDL is involved in atherogenesis, such as induction of adhesion protein expression and subsequent entry of mononuclear cells, formation of foam cells, and apoptosis and necrosis of vascular smooth muscle cells, inducing formation of the necrotic/lipid core [27]. Coronary endothelial dysfunction and inflammation are also associated with lipid-rich plaque or plaque morphology [28, 29]. Therefore, in the present study, the fact that only blood glucose variability correlated or tended to correlate with both markers of oxidative stress (urinary L-FABP values) and inflammation (hs-CRP values) as compared to conventional glucose indicators may attribute to its stronger association with coronary plaque instability.

When the culprit vessels excluding the culprit lesion (segment of 10-mm length centered at MLA site) were analyzed, a significant correlation of higher blood glucose variability with plaque instability was also found in the present study. Teraguchi et al. [13] have suggested that higher blood glucose variability is associated with coronary plaque vulnerability in the culprit lesion of AMI. In addition, Asakura et al. [30] previously reported the pan-coronary process of vulnerable plaque development in patients with MI. Our findings can add important insights to their findings: higher blood glucose variability links closely with both coronary plaques that lead to plaque rupture and non-culprit plaques.

### Conventional glucose indicators and lipid parameters

In contrast to previous IB-IVUS studies in which conventional glucose indicators (HbA1c, FPG, HOMA-IR) were associated with coronary plaque instability (increased lipid and decreased fibrous contents) [2, 3], the current study failed to show the significant association of these glucose indicators with plaque instability. In addition to the weaker correlations of sustained hyperglycemia with atherogenic factors discussed above, one possible reason for this discrepancy may be insufficient accuracy of conventional glucose indicators to measure the variety of glycemic disorder. Unlike the previous studies targeting patients with type 2 DM or impaired glucose tolerance alone [2, 3], our study population included various glycemic metabolism (normal glucose tolerance, impaired glucose tolerance, type 2 DM). HbA1c and FPG are mainly the indicators of chronic sustained hyperglycemia; therefore, the relative contribution of acute glucose fluctuation (postprandial hyperglycemia, hypoglycemia) to glucose disorder may not be exactly reflected in them [11, 31, 32]. Both postprandial hyperglycemia and hypoglycemia are important risk factors for cardiovascular disease, and have been reported to be associated with atherogenic factors, such as increased oxidative stress, inflammation and sympathetic nerve activity [33, 34]. Hence, glucose indicators reflecting both hyperglycemia and hypoglycemia, such as blood glucose variability, may be reasonable to evaluate glycemic disorder. The accuracy of HOMA-IR for predicting insulin resistance is also limited in subjects with higher FPG and lower beta cell function [35]. Thus, in this small population with various glycemic metabolism, conventional glucose indicators alone may not be sufficiently sensitive in reflecting the variety of glycemic disorder and thereby failed to show their significant association with coronary plaque instability.

Increased LDL-C value is a well-established risk factor for cardiovascular events and vulnerable plaques [36]; however, the present study failed to show significant correlation between LDL-C value and plaque instability. Several possible explanations for this may be proposed. First, our study included patients with both controlled (pre-treated with statins) and uncontrolled dyslipidemia, thereby potentially affecting our findings. Second, 49.1 % of the study patients had DM. Previous studies have shown that proportions of LDL particle size were different between patients with and without DM: small dense LDL (sd-LDL) particles are significantly higher in patients with DM [37]. Although we did not measure sd-LDL values, the assessment of LDL particle size may be able to reveal more clearly the link between dyslipidemia and plaque instability, because increased sd-LDL value is a stronger predictor for cardiovascular events compared to LDL-C value [38].

### Limitations

Several limitations should be noted in this study. First, this is a primarily hypothesis-generating, observational study with small sample size at a single center. Second, patients with severe glycemic disorder (i.e. patients treated with insulin) were excluded from this study. Although diabetes-related macrovascular disease can occur, even in the early stages of glycemic disorder, our findings will need to be confirmed in further investigations with larger sample size, including patients with severe glycemic disorder. Third, some patients were treated with anti-atherosclerotic drugs, such as statins and anti-diabetic drugs before admission. When analyzed separately, patients without pre-treatment with these drugs had similar results. While Kuroda et al. [10] have also reported the association between blood glucose variability and plaque instability in patients with CAD pre-treated with lipid-lowering therapy, the influence of these drugs should be sufficiently investigated in the future. Fourth, there may have been the potential influence of thrombus and vessel shrinkage on IVUS analyses because this study targeted culprit vessels of ACS. Fifth, increased lipid and decreased fibrous contents in the plaque have been reported to be associated with thin-cap fibroatheroma [23]; however, the association between blood glucose variability and fibrous cap thickness (another important measure to evaluate vulnerable plaque) remains unknown. Finally, it also remains unknown whether blood glucose variability in an acute phase and in a stable condition are similarly predictive for plaque instability. Furthermore, whether the assessment of intra- and inter-day blood glucose variability in a non-hospital setting could offer additional predictive value ought to be clarified.

## Conclusion

The current study revealed that compared to conventional glucose indicators (HbA1c, FPG, HOMA-IR), higher blood glucose variability as measured by CGMS was independently and more strongly associated with increased lipid and decreased fibrous contents with larger plaque burden and higher remodeling index in the culprit vessel of ACS patients. Our findings suggest that the assessment of blood glucose variability using CGMS may contribute to better prediction of vulnerable plaque, thus, blood glucose variability may become a new therapeutic target for the prevention of ACS in patients with coronary artery disease.

## Abbreviations

ACEI: angiotensin-converting enzyme inhibitor
AMI: acute myocardial infarction
ARB: angiotensin II receptor blocker
CAD: coronary artery disease
FPG: fasting plasma glucose
HOMA-IR: homeostasis model assessment of insulin resistance
MI: myocardial infarction
%FV: percent fibrous volume
%LV: percent lipid volume
